# The contribution of family physicians to surgical capacity at district hospitals in South Africa

**DOI:** 10.4102/phcfm.v13i1.3193

**Published:** 2021-10-27

**Authors:** Hans Hendriks, Adeloye Adeniji, Louis Jenkins, Robert J. Mash

**Affiliations:** 1 Harry Surtie Hospital, Northern Cape Government: Health, Upington, Northern Cape, South Africa; 2 Department of Family Medicine and Primary Care, Faculty of Medicine and Health Sciences, Stellenbosch University, Cape Town, South Africa; 3 Ceres Hospital, Western Cape Government: Health, Ceres, South Africa; 4 George Hospital, Western Cape Government: Health, George, South Africa

**Keywords:** district hospital, district health services, district health system, family physician, surgical capacity, surgery

## Abstract

The World Health Organization states that essential, cost-effective surgical care should be delivered at district hospitals. In South Africa significant skills gap exist at district hospitals, particularly in the area of surgery and anaesthesia. These small to moderate sized hospitals are too small to support a range of full time specialists even if they could be recruited and were cost-effective. Family physicians (FPs) are trained in the clinical skills required for district hospitals and primary health care. Clinical associates have also been introduced to perform procedures at district hospitals. This report illustrates the contribution of a FP to surgical care at Zithulele Hospital in the Eastern Cape. Family physicians not only bring the necessary clinical skills set but also increase the confidence and capacity of the whole team. Outreach and support by surgeons, as well as continuing professional development, are important. Surgical and anaesthetic skills must be developed together. Family physicians also bring leadership and clinical governance skills that ensure the inputs to support surgery, such as equipment and information systems are available. The contribution of FPs to surgery and district hospitals is overlooked in both policy and practice. Human resources for health policy should recognise their contribution and increase the numbers available and FP posts at district hospitals. There is also a need to update the package of emergency and essential surgical procedures in policy.

## Introduction

The World Health Organization states that essential, cost-effective surgical care should be delivered at district hospitals (DHs).^1^ In a review of surgical services in South Africa (SA) it was found that out of 327 public hospitals, 257 (79%) were district DHs, while the remaining 70 (21%) were regional and central hospitals.^2^ The DHs in SA are small to medium sized facilities, often situated in rural and even remote areas and usually the most accessible hospitals for local communities and for referrals from primary care. They are staffed by medical officers and most of them are involved in community service or are quite junior. It has been widely noticed that substantial skills gap exist at these hospitals, particularly in terms of essential surgical and anaesthetic services.^3,4,5^

These hospitals are usually too small and with too little surgery to support full time surgical and anaesthetic specialists. Organising DHs along traditional specialist disciplines is impractical, costly and inefficient. District hospitals are in essence generalist environments with male, female, maternal and child wards, and strong connections to the primary care platform. The clinical associates programme is one approach to increasing capacity to perform procedures at DHs, although numbers remain small and posts limited.^6^ Clinical associates must also work under supervision of a more senior doctor.

Family physicians (FPs), as specialists in family medicine are trained to work independently at DHs and in primary health care.^7^ Their training in well-functioning DHs provides a sufficient range of surgical and anaesthetic experience.^8^ Training can include rotations at regional or tertiary hospitals and outreach from these centres. Training requires the FP to become competent in a defined list of surgical and anaesthetic skills appropriate to the DH.^9^

In the Western Cape, it was the skills gap in DHs that prompted the head of health to support the emerging discipline of family medicine.^10^ Most DHs now have one or two FPs. Having a FP of course is not sufficient to fill these gaps without a team of other medical officers and the necessary equipment, supplies, medications, infrastructure, funding and health information. Family physicians must cover the whole hospital and support primary care. While previous work showed that the DH could provide the capacity for training in most surgical skills needed in the district health system,^8^ performing surgery is not the only responsibility of FP. Their roles also extend from being a consultant and clinician to leading clinical governance and building capacity in the team.^7^

In the following section, one of the authors recalls the difference that they made as a FP in the area of surgery at a DH.

## Surgery at a district hospital

After graduating as a FP and working at Ceres Hospital in the Western Cape, H.H. was looking for new challenges for himself and his family, so we moved to the Eastern Cape. H.H. worked at Zithulele Hospital, while training family medicine registrars and developing the family medicine programme for Walter Sisulu University. Zithulele Hospital is a 147-bed, 14-doctor hospital situated in one of the poorest communities in the country. It has very poor infrastructure and resources, a challenge that was mirrored in the healthcare system as a whole.

Before my (H.H.) arrival, the hospital only performed obstetric-based surgery, mainly caesarean sections and tubal ligations and uterine evacuations. Although the doctors had the skills to do other surgeries, confidence was low and time was limited. Most of the doctors developed niche areas of interest, for example, in tuberculosis (TB) and HIV-medicine, as both conditions contributed significantly to the burden of disease. A FP started work at Zithulele two years before my arrival. He became the champion for surgery, slowly but surely building capability and motivating for equipment, such as a functional anaesthetic machine.

My arrival boosted the support for surgery at Zithulele. Over time, we started conducting more surgery, in numbers and diversity. This was achieved by simply making time and supporting younger doctors to have the confidence to operate or give anaesthesia, teaching everyone who wanted to learn and by building a system where surgery could happen. With elective surgical lists and a new digitalised monitoring system in place, we could track how many cases we were doing, who was doing them and plan better for future procedures.

Having a digitalised information system was important to successfully motivate for equipment. Another important reason for monitoring performance closely was patient safety. Doctors need to learn correct and safe practice, something that usually only happens once you institutionalise a programme and do the clinical governance involved.

During my last year at Zithulele Hospital, we completed 966 cases in the one available theatre. All had theatre notes, surgical safety checklists and information on all these procedures were available digitally. Although two-thirds of the procedures were caesarean sections and tubal ligations, the diversity of the other third demonstrates how skills had grown: laparotomy for ectopic pregnancy, uterine evacuations, perineal repairs, dilatation and curettage, skin grafts, hydrocelectomies, circumcisions, orchidectomies with or without orchidopexy, amputations, fasciotomies and dentectomies for intellectually impaired persons. Of course, anaesthesia was needed for these cases, mostly spinal anaesthesia, but also general anaesthesia, conscious sedation and local blocks.

## Reflections on family physicians and surgery at district hospitals

To do surgery, especially in a DH setting, takes confidence and often quite a lot of guts. If you make a mistake, you are held responsible. As a result, especially in the current litigation prone era, doctors avoid surgery. There are many ways to boost confidence, none of them as effective as having a more experienced doctor next to you or at least on the premises. This is where the availability of a FP in settings where there is no surgeon, makes a big difference.

Other ways of boosting confidence is good outreach and support programmes from referral hospitals.^11^ Knowing they are there for advice and support when needed, helps a lot. Factors impacting successful outreach and support include the quality of relationships and communication, and planning site-specific models that work for all involved.^11^ Continuing professional development is also needed and there are more and more online programmes available such as the Incision Academy.^12^

Whether you are a senior doctor or the FP, one must always attempt to transfer skills to doctors and students with less experience. Every surgical opportunity should be considered as a chance to teach skills. Although family medicine has defined the surgical skills for training of FPs there is a need to update the package of emergency and essential surgical procedures in policy.^13,14^

Surgery comes with some form of anaesthesia. To be able to function in a DH setting, one needs to be comfortable with spinal and general anaesthesia, as well as some blocks (e.g. brachial block) and safe conscious sedation techniques. In this area, the FP, often by virtue of being older and more experienced, plays a critical role in transferring anaesthetic skills and giving doctors the confidence to do anaesthesia.

Family medicine teaches you to think bigger than just the immediate patient or procedure. You start thinking of a better booking system for surgical patients, how you can create elective surgical days, streamline admission and discharges on these days and follow-up systems. You plan and motivate for equipment to be able to work more effectively and to do more. You create monitoring and evaluation programmes to see if what you are doing is safe and effective, and if things go wrong to correct the problems. You start thinking of training staff, networking with your referral hospitals, developing healthy and effective referral pathways, building relationships and communicating in these networks.

Family physicians are repeatedly neglected in discussions and decisions involving the DH system. A recent ministerial task team, advising the government on human resources for health policy, completely overlooked the role of FPs in the DH.^15^ Likewise, surgeons debating on how to strengthen surgery in health districts often seem unaware of the contribution of FPs.^4^

The discipline of family medicine in SA has set a 10-year goal of one FP at every DH and community health centre or sub-district.^7^ Currently we have 0.16 FPs per 10 000 population and this varies from 0.3/10 000 in the Western Cape to 0.05/10 000 in Limpopo.^16^ Training programmes need 50–80 registrar posts each and each province needs to create five new family FP posts a year to achieve this initial goal.

## Conclusion

One of the major contributions that FPs can make to the health system is strengthening surgical and anaesthetic capacity at DHs. Surgery is not only about getting the procedure done, it is also about building the system around the procedure that makes it safe, cost-effective, evidence based and accessible to the patient. If we want to reach the goal of ‘enabling evidence-based, context-sensitive, safe surgical care, which is accessible, timely and affordable for all’, FPs are ideally suited to play a role because of the content and context of their training.
